# Effects of neuromuscular electrical stimulation on glycemic control: a systematic review and meta-analysis

**DOI:** 10.3389/fendo.2023.1222532

**Published:** 2023-07-31

**Authors:** Michael J. Sanchez, Ali Mossayebi, Solmaz Sigaroodi, Jehu N. Apaflo, Michelle J. Galvan, Kisuk Min, Francisco J. Agullo, Amy Wagler, Sudip Bajpeyi

**Affiliations:** ^1^ Metabolic, Nutrition, and Exercise Research (MiNER) Laboratory, Department of Kinesiology, The University of Texas at El Paso, El Paso, TX, United States; ^2^ Muscle Molecular Physiology Laboratory, Department of Kinesiology, The University of Texas at El Paso, El Paso, TX, United States; ^3^ Southwest Plastic Surgery, El Paso, TX, United States; ^4^ Department of Mathematical Sciences, The University of Texas at El Paso, El Paso, TX, United States

**Keywords:** e-stim, NMES, myostimulation, glucose, insulin sensitivity, muscle, metabolic health, substrate utilization

## Abstract

**Background:**

Physical inactivity increases the risk for metabolic diseases such as obesity and type 2 diabetes. Neuromuscular electrical stimulation (NMES) is an effective method to induce muscle contraction, particularly for populations with physical impairments and/or metabolic diseases. However, its effectiveness to improve glycemic control is unclear. This review aimed to determine the effectiveness of NMES on glycemic control.

**Methods:**

Electronic search consisted of MEDLINE (PubMed), EMBASE, Cochrane Library, Google Scholar, and Web of Science to identify studies that investigated the effects of NMES on glycemic control for this systematic review. The meta-analysis consists of the studies designed as randomized controlled trials. Effect sizes were calculated as the standardized mean difference (SMD) and meta-analysis was conducted using a random-effects model.

**Results:**

Thirty-five studies met the inclusion criteria for systematic review and of those, nine qualified for the meta-analysis. Existing evidence suggested that NMES effectively improves glycemic control predominantly in middle-aged and elderly population with type 2 diabetes, obesity, and spinal cord injury. The meta-analysis is comprised of 180 participants and reported that NMES intervention lowered fasting blood glucose (SMD: 0.48; 95% CI: 0.17 to 0.78; p=0.002; I²=0%). Additional analysis using the primary measures reported by each study to indicate glycemic control (i.e., OGTT, HOMA-IR, and fasting glucose) also confirmed a significant effect of NMES on improving glycemic control (SMD: 0.41; 95% CI, 0.09 to 0.72; p=0.01; I²=11%). NMES protocol varied across studies and requires standardization.

**Conclusion:**

NMES could be considered as a therapeutic strategy to improve glycemic control in populations with physical impairments and/or metabolic disorders.

**Systematic review registration:**

https://www.crd.york.ac.uk/prospero/, identifier CRD42020192491.

## Introduction

Physical inactivity increases risk for metabolic diseases such as insulin resistance, obesity, type 2 diabetes (T2D) and is the fourth leading risk factor for death worldwide (1–6). Physical inactivity-related diseases contribute to a heavy economic burden through direct health care related expenditures, indirect productivity costs, lifetime disease burdens, as well as premature mortality (7). Adhering to the Centers for Disease Control and Prevention recommended physical activity guidelines (150 min/week) could avert the increasing cases of metabolic disorders such as diabetes and may decrease the financial burden on the health care system (8, 9). A prominent metabolic disorder evident in diabetes is the loss of glycemic control capability (10). It is well established that muscle contraction through endurance and resistance exercise is effective in improving insulin sensitivity in all populations (11–13). Muscle contraction induced by electrical stimulation in human muscle cells (*in-vitro*) as well as in isolated rat skeletal muscle have been shown to upregulate glucose uptake (14, 15). Therefore, as an alternative therapeutic approach, the potential to improve glycemic control by inducing muscle contractions through electrical stimulation is of particular interest to populations who are less likely or unable to perform regular physical activity or who are insulin resistant.

Neuromuscular electrical stimulation (NMES) is an alternate strategy to induce involuntary contraction of skeletal muscle via depolarization of the motor axons and nerves being stimulated through an electrical current (16–19). NMES has been widely used across the field of rehabilitation to prevent muscle loss, regain muscle mass and function, and to improve motor learning and exercise performance in individuals with spinal cord injury (SCI), stroke, sport-related injuries, as well as individuals with metabolic diseases (20–26). Specifically, previous studies have established the effectiveness of NMES in preventing muscle loss, improving skeletal muscle mass, power, and work capacity following SCI (23, 27), improving muscle strength in rodents and humans (17, 24, 25), and both knee function and quadricep strength following cruciate ligament reconstruction in humans (25). Use of NMES has also been reported to increase skeletal muscle cross sectional area and capillary number per type IIA and IIB muscle fibers (27–31). Voluntary muscle contraction recruits motor units in an orderly fashion established as the size principle (32, 33), and recruits’ type I fibers under low intensity exercise (33, 34). NMES on the other hand, has been shown to recruit motor units in a reverse pattern. Electrical stimulation preferentially recruits motor units within the spatial area of the electrode and also motor units with greater excitability, larger axonal diameter, and lower resistance against external electrical stimulation (18, 35). It has been suggested that an increase in glucose uptake with involuntary muscle contraction induced by electrical stimulation is due in part to the preferential activation of glycolytic type II fibers (28, 36, 37). This is also supported by rodent studies reporting preferential recruitment of axons with larger diameters and fibers that are more reliant on glycolytic metabolism through NMES (38–42). It has been shown that spatial muscle recruitment with NMES is associated with electrode placement, stimulating the motor neuron branches that are in the proximity of the electrical current being delivered (43). Conversely, muscle fiber type determines the recruited motor units in voluntary contraction (43). Electrical stimulation leads to activation of glycogenolysis and anaerobic glycolysis as the major source of ATP production (44). Translocation of glucose transporter (GLUT-4) to muscle membrane (39–41, 45) and increase in glucose uptake (46–48) have been reported with muscle contraction that uses an insulin independent glucose uptake pathway. An increased accumulation of lactate (44, 49) and whole-body carbohydrate utilization (44, 50, 51) have been reported during electrical stimulation. Similarly, a higher Pi/PCr ratio and lower intracellular pH causing early fatigue (exaggerated metabolic demand) have been reported during high frequency electrically stimulated muscle contraction compared to voluntary contraction (34, 43).

Skeletal muscle, being the primary site for insulin stimulated glucose uptake, plays an important role in glycemic control and regulation of whole-body glucose metabolism (45, 48). Electrically inducing skeletal muscle contractions as a way to improve glucose utilization has been used in a surgical setting to acutely prevent a hyperglycemic response during preoperative anesthesia (52). However, existing literature that assessed the effectiveness of NMES in improving glycemic control and metabolic health is not conclusive. This gap in knowledge is due to highly variable NMES protocols used (frequency, duration, and length of intervention), population studied, variable testing methods used to access glycemic control, and lack of control group in several studies. Therefore, the primary purpose of this comprehensive systematic review and meta-analysis was to evaluate the existing evidence to determine the effectiveness of NMES as an alternative therapeutic approach to improve glycemic control. As improvements in glycemic control have often been connected to whole body substrate utilization and lean mass, we have therefore also explored the existing literature to determine the effects of NMES on substrate utilization and body composition.

## Methods

### Electronic search strategy and eligibility criteria

This systematic review and meta-analysis were performed in accordance with the Cochrane Collaboration (53) and Preferred Reporting Items for Systematic Review and Meta-analysis (PRISMA) guidelines (54). The protocol of the study was registered on International Prospective Register of Systematic Review (PROSPERO) (CRD42020192491). Randomized controlled trials that evaluated the effects of NMES on glycemic control and/or insulin sensitivity were included. A computerized search was performed on MEDLINE (PubMed), EMBASE, Cochrane Library, Google Scholar, and Web of science to identify all potential literature. Various combinations of keywords and mesh words relating to neuromuscular electrical stimulation were used in the search **(**See Table, **Supplementary Table 1**, which illustrates the keyword/meshword search strategies used). References of selected studies were further reviewed to include any additional studies that may not have been found through search terms. The search was not restricted to any geographical region, gender, or population, but was restricted to studies published in English language and conducted on human subjects.

### Study selection

In the initial search, four researchers (MS, MG, AM, SS) independently located and reviewed all articles by title and abstract text to ensure that the following inclusion criteria were met for the systematic review: 1) studies administered neuromuscular electrical stimulation on skeletal muscle, 2) articles reported data from original research and did not include secondary data, 3) articles reported glycemic control data and 4) studies were conducted in human subjects. Studies that met inclusion criteria for the systematic review were then considered for meta-analysis if the following additional criteria were met: 1) studies were conducted with a placebo or equivalent control group and 2) articles presented both pre and post NMES intervention data for primary outcome measures with mean and standard deviation or standard error of mean values. All reviewers assessed the selected articles and collectively resolved any discrepancies for initial inclusion. After the potential articles were identified based on the initial criteria, a full text review of all articles was performed before proceeding to data extraction.

### Data collection/extraction

Authors independently extracted all relevant data needed for both systematic review and meta-analysis. Extracted data included characteristics of participants (age, gender, body mass index (BMI), and health status), sample size, intervention type (acute or chronic), anatomical location of NMES application, NMES application protocols (frequency, intensity, duration, and length of intervention), testing methods used to assess glycemic control, and effects of NMES on glycemic control (acute and chronic effects of NMES), substrate utilization, and body composition. Meta-analysis was limited to analyzing the effects of NMES on glycemic control using only longitudinal studies that met inclusion criteria (n=9). Due to the limited number of studies that met the inclusion criteria, it was not achievable to conduct a meta-analysis to determine the effects of NMES on substrate utilization (n=1) and body composition (n=4). Following the data extraction phase, all reviewers verified entered data to confirm the accuracy.

### Risk of bias and quality assessment

Reviewers independently assessed the risk of bias for the studies included in meta-analysis using the Cochrane Collaboration’s Risk of Bias tool (RoB_2_) (55). Studies were assessed for the following criteria: random sequence generation, allocation concealment, blinding participants, blinding of outcome assessment, incomplete data reporting, and selective reporting.

### Data analysis

The meta-analysis was carried out to determine the effects of NMES on glycemic control, the primary outcome measure of the study. Continuous outcomes were reported as the mean difference (MD) and standardize mean difference (SMD) from pre to post treatment in each group with 95% confidence interval (95% CI). Random effect models were used to combine data in Review Manager (version 5.3). The statistical heterogeneity among studies was tested using I² statistics. I² values 25-50% were considered indicative of low heterogeneity, 50-75% were considered moderate heterogeneity and values above 75% were considered to have a high degree of heterogeneity. A *p* value < 0.05 was considered statistically significant.

## Results

### Study selection

**Figure 1:**
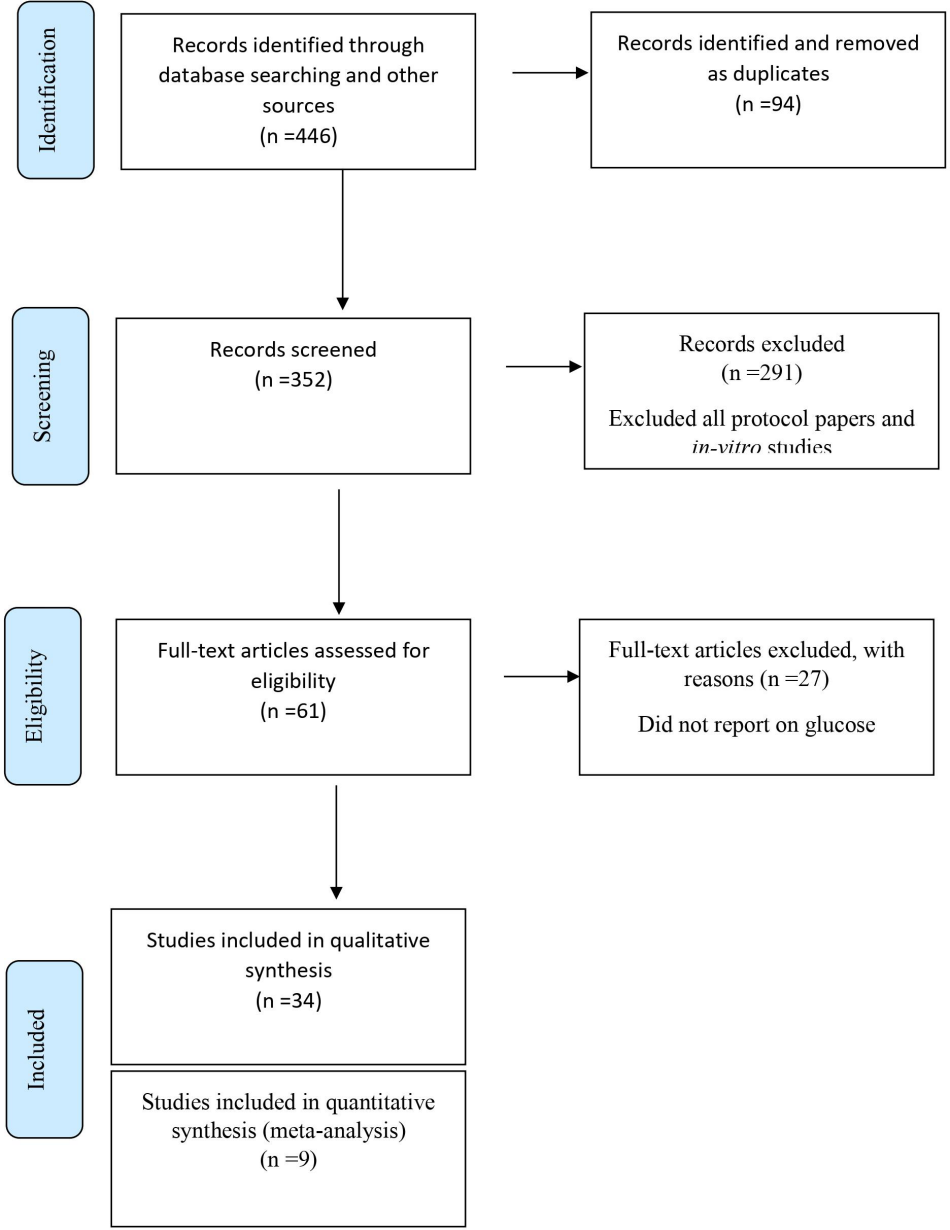
Flow diagram of search strategy.

The PRISMA flow diagram details the database search results along with all exclusion rationale (**Figure 1**). Of the 446 original identified studies through the database search, 385 studies were excluded for having been identified as a duplicate, protocol paper, *in-vitro*, or animal studies. Of the remaining 61 studies, 27 studies were removed after a thorough full-text assessment revealed that studies did not report on primary outcome measures. The remaining 34 studies met the inclusion criteria for the systematic review, while nine randomized controlled longitudinal studies met inclusion criteria for the meta-analysis.

### Population characteristics

Population characteristics of the reviewed studies are described in Table, **Supplementary Table 2**. The 34 studies in this systematic review were conducted on healthy individuals (n=7) and populations with obesity (n=3), T2D (n=14), SCI (n=9), and cystic fibrosis (n=1). Data from these 34 studies consisted of a total of 527 young, middle age, and elderly healthy weight, obese, population with T2D or SCI where sample size in each intervention study ranged from 5-75. Among all included studies, 20 studies included male and female subjects whereas 12 studies were conducted on only male subjects and two studies were conducted on only female subjects. Nine randomized controlled studies included in the meta-analysis consisted of a total of 180 young healthy weight, obese, population with T2D or SCI where sample size in each intervention ranged from 9-46. In the meta-analysis a total of 91 participants were allocated to NMES group while 89 participants were allocated to control/placebo group.

### Study design and methods used to measure primary outcome

Study characteristics and testing methods used to measure glycemic control, substrate utilization, and body composition in all studies included in this systematic review are outlined in Table, **Supplementary Table 2**. Thirteen studies reported on acute effects and 22 studies reported on chronic effects of NMES, with one of those studies reporting on both acute and chronic effects of NMES. Studies reported one or multiple measures of glycemic control/insulin sensitivity which included the fasting blood glucose (n=17) (30, 50–52, 56–68), fasting insulin (n=11) (23, 31, 50–52, 60, 61, 66, 69–71), homeostatic model assessment index (HOMA-IR) (n=4) (50, 52, 60, 70), Matsuda index (n=1) (72), oral glucose tolerance test (OGTT) (n=11) (23, 29, 31, 45, 69–75), meal glucose tolerance test (MGTT) (n=1) (73), HbA1c (n=5) (50, 58, 64, 69, 76), and hyperinsulinemic euglycemic clamp (n=6) (69, 71, 75–78). Among all longitudinal studies that met the inclusion criteria for meta-analysis (N=9) reported fasting blood glucose before and after NMES intervention. Additionally, other relevant insulin sensitivity measures were also reported. This includes fasting insulin (n=4) (30, 72, 73, 79), HOMA-IR (n=3) (30, 72, 79), Matsuda index (n=1) (72), MGTT (n=1) (73), OGTT (n=2) (72, 74), Glucose area under the curve (AUC) (n=3) (72, 74, 79), Insulin area under the curve (AUC) (n=2) (72, 79), and Glycated Hemoglobin (HbA1c) (n=2) (30, 62).

### Overview of neuromuscular electrical stimulation protocol

The NMES protocols used in included studies are outlined in Table, **Supplementary Table 2**. This includes information reported on length of NMES interventions, number of sessions, duration of sessions, frequency, and intensity. NMES frequency below 50 Hz has generally been considered as a low frequency (45, 49, 77), and a frequency of 50 Hz or above is considered as high frequency (49, 70, 74, 80, 81) in existing literature. Therefore, alongside presenting the specific frequency reported in articles, we have also reported frequency as “low” or “high”. Twenty-three studies used low frequency, eight studies used high frequency, two studies reported using both low and high frequency, while one study did not specify the selected frequency for NMES application. Although most studies reported on frequency and duration, NMES intensity was inconsistently reported across the studies. Most of the studies reported intensity as up to maximum tolerable levels (n=14), whereas some studies reported a range from 5-140 mA (n=8). Maximum tolerable intensity level usually varies from one person to another (82). One study reported intensity by oxygen consumption, and eleven studies did not report on NMES intensity. Most studies ranged from 2-8 weeks in duration of NMES intervention. Among the nine studies included in meta-analysis, four studies used low frequency, four studies used high frequency, while one study used both low and high frequency for NMES application. Four studies reported intensity as up to maximum tolerable level, one reported intensity at 60 mA, one study reported intensity at 5-10 mA, and three studies did not report NMES intensity. Duration of NMES session and length of NMES intervention also varied among studies included in meta-analysis. Majority of the studies reported session times ranging from 5-40 minutes with the most common intervention duration lengths of 2-8 weeks.

### Risk of bias

**Figure 2:**
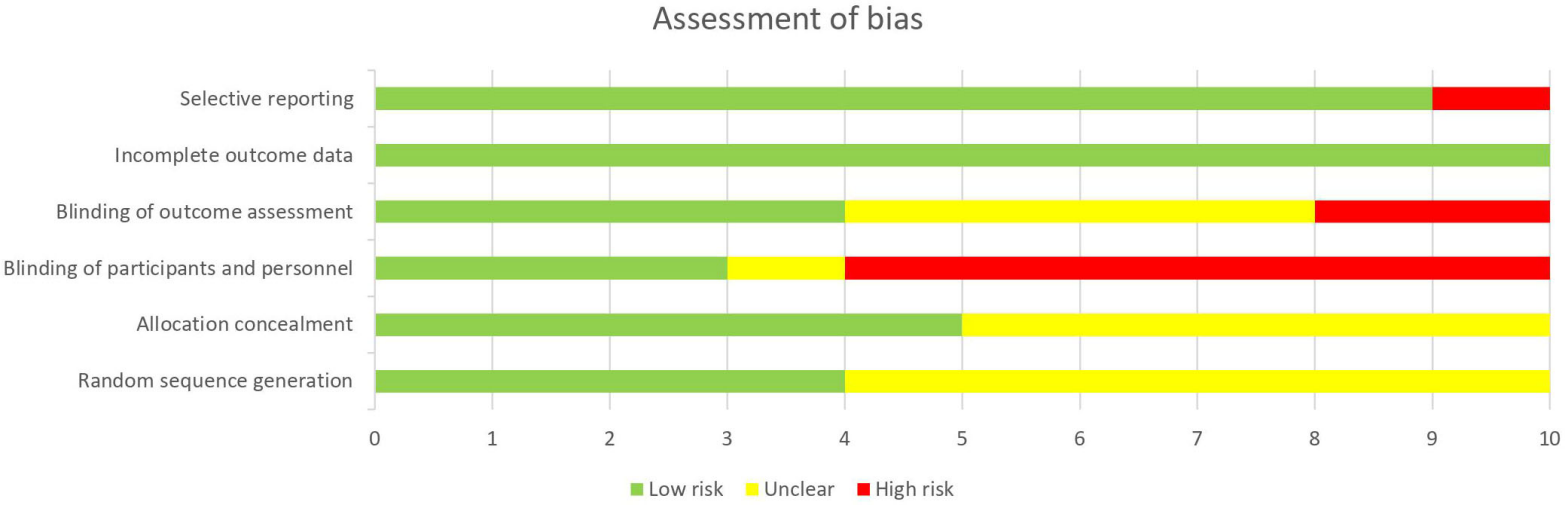
Assessment of bias (percentage) for studies included in meta-analysis.

**Figure 2** summarizes the assessment of quality and risk of bias of the studies. All reviewers used the Cochrane Collaboration’s risk of bias (RoB) tool to evaluate each study for risk of bias. Risk of bias assessment reported an overall outcome of low to moderate.

### Outcome of included studies

#### Acute effects of NMES on glycemic control

Among 34 studies included in this systematic review, 13 studies investigated acute effects of NMES on glycemic control (including a study reporting both acute and chronic effects) in populations with hyperglycemia and T2D (n=5), obesity (n=1), as well as in a healthy population (n=7). For the population with Obesity or T2D, all studies reported NMES being effective at acutely improving glycemic control. In a healthy population, five (52, 67, 68, 77, 78) out of the seven studies reported an improvement in glycemic control. Overall, six studies (45, 50–52, 61, 66) reported a significant decrease in blood glucose and three studies reported an increase in glucose disposal measured during hyperinsulinemic euglycemic clamp (76–78) with acute application of NMES. Overall, present evidence strongly indicates increased glucose utilization during NMES application.

#### Chronic effects of NMES on glycemic control

There were 22 longitudinal studies that investigated the chronic effects of NMES on glycemic control were included in this systematic review. Except for four studies that investigated young adult population (30, 59, 69, 74), all studies were conducted in middle-aged and elderly men and women. Majority of the studies (n=16) reported improvement in glycemic control measured by various methods including fasting blood glucose (56–58, 60, 62, 63), OGTT (23, 29, 31, 72, 79), MGTT (73), HbA1c (64, 70), and hyperinsulinemic euglycemic clamp (75, 76), while two studies reported no changes in glycemic control as measured by fasted blood glucose (65), and hyperinsulinemic euglycemic clamp (71).

#### Meta-analysis

**Figure 3:**
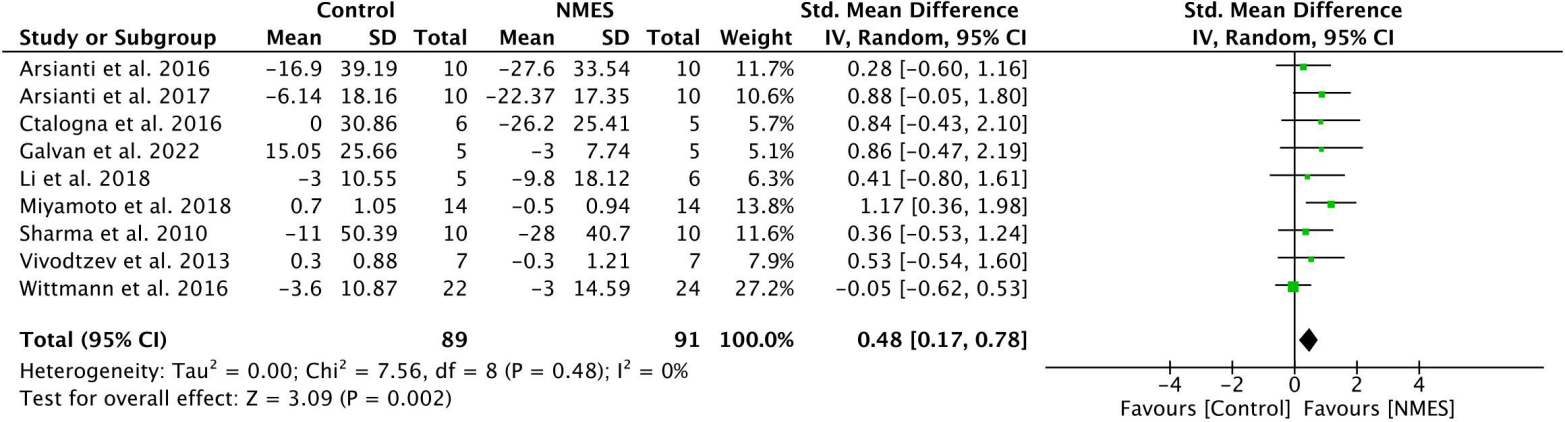
Forest plot indicating effects of NMES on fasting blood glucose.

Nine longitudinal studies met the inclusion criteria for a meta-analysis to examine the effectiveness of NMES on glycemic control, measured by fasting blood glucose (**Table 1**). The meta-analysis determined a significant effect of NMES on lowering fasting blood glucose (SMD: 0.48; 95% CI: 0.17 to 0.78; p=0.002; I²=0%) (**Figure 3**). The methods used to assess glycemic control varied among studies. Two studies (72, 74) used OGTT, one study used HOMA-IR (30) whereas rest of the studies used blood glucose (56, 57, 62, 63, 65, 73) to assess glycemic control. Additionally, one study (79) that met all the inclusion criteria and reported glycemic control measured by OGTT but did not report fasting glucose level before and after the NMES intervention. Therefore, we performed additional analysis (n=10) using the data from primary measures reported by each study to indicate glycemic control (i.e., OGTT, HOMA-IR, and fasting glucose). This analysis also indicated a significant effect of NMES on improving glycemic control (SMD: 0.41; 95% CI: 0.09 to 0.72; p=0.01; I²= 11%) (See Figure, **Supplementary Figure 1**, a forest plot indicating effects of NMES on glycemic control).

**Table 1:** Population characteristics of studies included in meta-analysis.

Study	Number of participants	Study population	NMES Intervention	NMES duration (min)	NMES frequency (Hz)	NMES pulse width (µs)	NMES Intensity	Method to measure IS	Glycemic control outcome	Body composition (Methods)	Substrate utilization (Methods)
**Arsianti et al. (** 56 **), (a)**	N=20 NMES=10 Passive Stretching=10	Men and women with T2D (≥55 years old)	3x/week for 4 weeks	30	Low 20	200	60 mA	BG	BG: Decreased	Not measured	Not measured
**Arsianti et al.** (57) **(b)**	N=20 NMES=10 Control=10	Men and women with T2D (≥35 years old)	3x/week for 4 weeks	30	Low 20	200	NA	Random BG	BG: Decreased	Not measured	Not measured
**Catalogna et al.** (73)	N=11 NMES=5 Control=6	Men and women with T2D (45-75 years old)	7x/week for 2 weeks	5	Low 1.33/burst mode of 16	150	5-10 mA	MGTT	BG: Decreased Postprandial Glucose: Decreased	Not measured	Not measured
**Galvan et al.** (74)	N=10 NMES=5 Control=5	Overweight/. obese men and women (18-54 years old)	3x/week for 4 weeks	30	High 50	300	Max tolerable	OGTT	BG: No change Glucose AUC: Decreased	BW: No change BMI: No change FM: No change LM: No change (DXA)	RQ: No change (Indirect Calorimetry) Lactate: Increased
**Li et al.** (72)	N=11 NMES=6 High protein diet=5	Middle-aged men and women with SCI (37-58 years old)	3x/week for 8 weeks	30	High 50	450	NA	OGTT	BG: Decreased Insulin AUC: Decreased Fasting insulin: No change Matsuda Index: No change HOMA-IR: No change Glucose AUC: No change	Body Mass: Decreased FM: Decreased LM: No change Android Fat Mass: Trend for Decrease (DXA)	Not measured
**Miyamoto et al.** (62)	N=28 NMES=14 Control=14	Elderly men with T2D (60.2-66.2 years old)	5x/week for 8 weeks	40	Low 4	0.2	Max tolerable	Fasting BG	BG: Decreased HbA1c: No change	BW: No change BMI: No change %BF: Decrease LM: No change (BIA)	Not measured
**Sharma et al.** (63)	N=20 NMES=10 Control=10	Men and women with T2D (>55 years old)	3x/week for 2 weeks	40	High 50	NA	Max tolerable	Fasting BG	BG: Decreased	Not measured	Not measured
**Vivodtzev et al.** (30)	N=14 NMES=7 Control=7	Men and women with Cystic fibrosis (21-43 years old)	4x/week for 6 weeks	30	Low 35 for 2 weeks followed by High 50 for 4 weeks	400	Max tolerable	Fasting BG	BG: Decreased HOMA-IR: Decreased	Mid-Thigh circumference and quadricep strength: Increased	Not measured
**Wittmann et al.** (65)	N=75 NMES=24 NMES+Diet=21 Control=22 Drop out=8	Elderly women with Sarcopenic obesity (≥70 years old)	1x/week for 26 weeks	11-20	High 85	350	Borg rates of perceived exertion of 5-6 on a 10 point scale	Fasting BG	BG: No change	Waist Circumference: Decreased (DXA)	Not measured

NMES, Neuromuscular electrical stimulation; IS, Insulin sensitivity; T2D, Type 2 diabetes mellitus; SCI, Spinal cord injury; OGTT, Oral Glucose Tolerance Test; MGTT, Meal Glucose Tolerance Test; HOMA-IR: Homeostatic Model Assessment of Insulin Resistance; HbA1c, Glycated Hemoglobin; BG, Blood glucose; BW, Body weight; FM, Fat mass; BF, Body fat; LM, Lean mass; DXA, Dual energy X-ray Absorptiometry; BIA, Body Impedance Analysis; RQ, Respiratory Quotient; NA, Not Applicable; Min, Minute; Hz, Hertz; µs, Microsecond.

#### Effect of NMES on substrate utilization

To our knowledge seven studies have reported the acute effects of NMES on substrate utilization measured by Respiratory Quotient (RQ) or Respiratory Exchange Ratio (RER), oxygen consumption (VO2), and lactate production. All these studies indicate increased glucose utilization during NMES application as measured by increased RQ (50, 51, 77, 78, 83, 84), increased lactate level (50, 51, 61, 77, 78) or elevated oxygen utilization (50, 51, 77, 78, 83) (see Table, **Supplementary Table 2**). Additionally, three studies reported on energy expenditure during NMES. Two of these studies reported an increase (69, 84), and one study reported no change (76) in energy expenditure. Only one study, to our knowledge, investigated the chronic effects of NMES on substrate utilization (74) and reported no change in resting substrate utilization and energy expenditure after four weeks of NMES.

#### Body composition

Among all studies that met inclusion criteria for this systematic review, nine studies reported on body composition parameters at baseline and at the end of the NMES intervention (see Table, **Supplementary Table 2**). Six studies (23, 58, 72, 74, 75, 79) used dual-energy X-ray absorptiometry (DXA), and three studies (62, 64, 69) used bio-electrical impedance assessment (BIA) to assessed body composition. No significant changes in body composition were reported in majority of the studies (64, 69, 74, 79). Two studies (58, 60) reported a significant reduction in total body weight and body fat without any changes in lean body mass after 8 and 12 weeks of NMES intervention. One study reported significant increase in body mass and lean muscle mass (23) and one study (62) reported a significant decrease in body fat without any change in body weight and lean mass after NMES treatment. One study (72) that combined NMES with exercise reported a significant decrease in body mass and fat mass, as well as a trend for decrease in android fat mass.

## Discussion

The aim of this systematic review and meta-analysis was to investigate the effects of NMES on glycemic control. Based on the existing evidence and meta-analysis, we conclude that acute application of NMES is effective in improving glucose utilization, while chronic use of NMES is effective in improving glycemic control particularly in populations with physical impairments and/or metabolic disorders such as T2D, obesity, and/or SCI.

NMES is an alternate strategy to induce skeletal muscle contraction and has been widely used in rehabilitation settings to prevent muscle atrophy and loss of muscular strength (21, 23, 27, 29, 85). Equivalent to the effect of an action potential seen in voluntary muscle contraction, the electrical current from NMES also results in changes to the membrane potential, which in turn releases calcium and initiates the signaling cascade leading to skeletal muscle contraction. The energy requiring mechanism that is seen in voluntary muscle contraction is also seen with the use of NMES, thus implying a possible beneficial effect by its potential to increase energy expenditure (69). Elevated ATP utilization has been demonstrated in skeletal muscle after intermittent NMES application (86). Interestingly, a greater anaerobic ATP turnover has been reported with electrically induced contractions, compared to equivalent voluntary contractions of the soleus and gastrocnemius muscles (87). Several studies indicated use of glycolytic sources as major substrates during NMES induced muscle contractions (49, 56–58, 73, 74). Increased glucose uptake has been shown with electrical stimulation using cell culture model (88) as well as in isolated rat skeletal muscle (47).

Downregulation of insulin dependent glucose uptake pathway has often been reported in upstream signaling molecules such as insulin receptor substrate 1, phosphatidylinositol 3-kinase, and Akt phosphorylation (89) with little to no impact on GLUT4 content and GLUT4 translocation in population with insulin resistance and T2D (90). Increase in GLUT4 content and GLUT4 translocation to cell membrane are well established mechanisms to increase glucose uptake during exercise in healthy and people with T2D (90, 91). Muscle contraction induced by electrical stimulation has also been shown to effectively increase AMP-activated protein kinase-α, Ca^+2^/calmodulin-dependent protein kinase II CaMKII and Akt phosphorylation (89), upregulate GLUT4 content and translocation (31), deplete muscle glycogen (80), increase glucose uptake from peripheral circulation (45, 50–52, 61, 66, 77, 78) and increase whole body glucose utilization (50, 51, 61, 74, 77, 78). Therefore, NMES seems to have great potential to be used as an effective alternate strategy to improve glycemic control especially in physically inactive population with insulin resistance and T2D via insulin independent glucose uptake pathway.

### Acute effects of NMES on glycemic control

Existing evidence strongly suggests that NMES application utilizes carbohydrates as fuel and acutely increases glucose utilization in healthy, T2D, and SCI populations. Several studies reported a decrease in blood glucose level (45, 50–52, 59, 61, 66–68, 76–78), an increase in whole body glucose utilization as measured by rise in RQ (50, 51, 77, 78), increased lactate production (50, 51, 67, 77, 78), and elevated glucose uptake as measured by hyperinsulinemic euglycemic clamp (76–78) with NMES application. In addition to an increased glucose disposal rate, Hamada et al. demonstrated maintaining this elevated glucose uptake for at least 90 minutes following the cessation of NMES application, which also showed a greater requirement for glucose during the poststimulation period (77). Other studies, however, did not observe any significant acute effect of NMES on postprandial glucose level among healthy active participants (69, 83, 84). Combining whole body NMES with voluntary exercise has also shown an additive effect in elevating RQ and increasing blood lactate concentration (92). Holzer et al. however reported no significant additive effect of whole body NMES with resistance training on postprandial glucose measured by continuous glucose monitor (CGM) (93), a device. Additionally, increased Akt phosphorylation which is associated with contraction-induced GLUT4 translocation signaling mechanism (89) and recruitment of predominately glycolytic type II muscle fibers (36) have also been reported during NMES. These findings suggest the potential of NMES to maintain and enhance the function of type II muscle fibers in glucose uptake independent of insulin signaling. Further studies are needed to fully elucidate the exact mechanism by which NMES impacts blood glucose levels and glucose disposal rates in both healthy individuals and people with dysglycemia. Most of the above-mentioned studies reported increases in glucose utilization regardless of NMES intensity (low or high) and duration.

Effectiveness of NMES on glucose utilization was evident in healthy as well as population with T2D. However, studies in young and healthy/obese populations are limited and it is unknown if NMES could acutely affect blood glucose levels in these populations. Most studies in this review used low frequency stimulation. Only three of the studies; Poole et al. (50 Hz) (69), Wall et al. (60 Hz) (66) and Guzman et al. out of the 13 studies applied high stimulation frequencies. Wall et al. reported a decrease in blood glucose with no change in insulin level while Poole et al. study showed no significant change in neither blood glucose nor glucose disposal rate, with both studies involving small sample sizes of 6 and 5 respectively. Guzman et al. reported a significant hypoglycemic effect of 5 Hz contrary to higher frequencies (10 Hz and 50 Hz). Future investigation should determine optimum frequency, intensity, and duration for NMES that could be beneficial for populations with hyperglycemia and insulin resistance.

### Chronic effects of NMES on glycemic control

To the best of our knowledge, this is the first systematic review with meta-analysis to investigate the effectiveness of NMES on chronic glycemic control. The majority of the studies in this systematic review indicated acute and chronic improvements in glycemic control after NMES interventions. Meta-analysis with randomized controlled trials strongly suggests that NMES can be used effectively as an alternative strategy to improve chronic glycemic control. It should be noted that longitudinal studies that met inclusion criteria for the meta-analysis, were limited to populations with obesity, T2D, SCI, and cystic fibrosis. Future investigation should focus on studying the effectiveness of NMES in normoglycemic populations. Methods used to assess glycemic control also varied across the studies. However, all but one study (79), reported on fasting blood glucose before and after the NMES intervention. A significant decrease in fasting blood glucose was reported by all studies, with the exception of Wittman et al. (65) and Galvan et al. (74). This lack of improvement in the Wittman et al. study may be due to the NMES parameters prescribed which included treatment once a week for 26 weeks at an inconsistent intensity. Furthermore, the elderly female participants in this study had sarcopenic obesity which is characterized by a progressive loss of muscle mass and a high fat mass which may explain why an improvement in glycemic control was not reported in this particular population. Galvan et al. study was conducted in people without diabetes with a small sample size which might be the reason why no improvement in fasting blood glucose was observed. Their study however, showed improvement in glucose tolerance. Specific NMES protocol should be further investigated to determine the effectiveness of NMES in this special population. Studies that met the inclusion criteria for meta-analysis, also used various methods to measure insulin sensitivity (OGTT, HOMA-IR, and fasting glucose). It is unknown whether the methods used to assess insulin sensitivity in the studies impacted the ability to detect changes in glycemic control brought about by NMES. However, as our analysis strongly suggest an impact of NMES on reducing fasting glucose, it can be expected that the effectiveness can be confirmed using more sensitive methods to measure insulin sensitivity. With the exception of Wittman et al. (65), all studies in the meta-analysis reported favoring NMES as an effective intervention in improving glycemic control, regardless of frequency (low or high), varied session times, duration and intensity. Therefore, NMES intervention was shown to be an effective alternative strategy to improve glycemic control in population with metabolic diseases and mobility limitations.

### Effects of NMES on substrate utilization and body composition

Although the primary purpose of this review was to determine effects of NMES on glycemic control, we also explored the effects of NMES on whole body substrate utilization and body composition. A greater reliance on whole body fat oxidation and metabolic flexibility has been well established with insulin sensitivity (94). RER has been shown to increase with exercise intensity and has been referred to as an indirect method of revealing the oxidative capacity of skeletal muscle (68, 93). Lower RER values and higher oxidative metabolism has been observed in trained males when compared to untrained males at similar submaximal workloads (67). Given skeletal muscle is the largest site for insulin stimulated glucose uptake and has been associated with insulin sensitivity (23, 29, 79), we aimed to determine if NMES is also effective in improving whole body substrate utilization and body composition. There were only six studies that reported on whole body substrate oxidation during acute use of NMES, measured by indirect calorimetry (50, 51, 61, 74, 77, 78). These studies indicated increase in whole body carbohydrate utilization during NMES. Two other studies reported an increase in whole body carbohydrate utilization using a Douglas Bag method (84) while Cohen et al. showed an additive effect of NMES with blood flow restriction on carbohydrate utilization (83). An increased whole body oxygen uptake measured by RER has been noted at the onset of low frequency electrical stimulation, followed by an immediate return to resting levels at the termination of stimulation (77, 78). There is lack of longitudinal studies that assessed effects of chronic use of NMES on whole body substrate utilization. To the best of our knowledge, there is only a pilot study that investigated the chronic effects of NMES on whole body substrate utilization and reported no effects from NMES (74). Effects of chronic use of NMES on body composition is also limited. Most studies indicated that there was either no change in body composition or no gain in lean muscle mass (62, 72, 74).. NMES has been shown to increase lean body mass, as well as muscle force and strength when stimulated at a frequency of at least 50 Hz in patients with SCI (23). Overall, the limited data indicates no change in body composition after NMES use with some indication of improvement in muscle mass in patients with SCI. Future studies should investigate long term effects of NMES sessions on substrate utilization and body composition to understand if the NMES induced improvement in glycemic control can be achieved independent of concurrent improvements in substrate utilization and/or muscle mass. Future studies should also consider evaluating the effects of NMES on other cardiovascular health aspects such as blood flow, breathing rate, oxygen saturation, heart rate, and blood pressure to fully understand the implication of NMES on various components of cardiovascular health.

### Recommended NMES protocol to improve glycemic control

Although our findings strongly suggest the effectiveness of NMES to improve glycemic control, a specific recommendation of NMES protocol has not been established. Lack of randomized controlled trials along with varied study populations make this challenging to determine an effective recommendation. Present literature indicates both low and high intensity NMES with varied frequency has been effective in acutely increasing glucose utilization as well as improving insulin sensitivity. While considering the NMES protocol, it is important to recognize safety and comfort of the individuals, and the target population (e.g., insulin resistant or having physical limitations to perform physical activities etc.). The side effects and discomforts with NMES use are not clearly described in most studies. While some studies indicated that participants did not report significant pain with NMES use (45, 95), physical discomfort, pain, or low subjective tolerance were reported in few studies (96–99). This is particularly seen with high frequency and high intensity, which is also known to increase muscle fatigue (100). A higher NMES intensity has been shown to result in a greater glucose uptake when compared to lower intensities (45). Significant correlations between stimulation intensity and blood glucose levels revealed that the contraction intensity substantially contributes to acute glucose metabolism (45). Poor tolerability is often explained as a limiting factor in many previous studies. Majority of the reviewed studies used a maximum tolerable intensity, which indicates a subjective intensity that varied across studies and participants. The option for the participants to select a maximum tolerability has been shown to lead to better compliance but less consistency with the given intervention. Tolerability and compliance should be considered when developing an NMES intervention. Therefore, it has been suggested that a low frequency NMES can be employed in attempt to minimize subject discomfort while still achieving efficiency and largely activating glycolytic type II muscle fibers that substantially utilize carbohydrate and glycogen, while improving insulin sensitivity (36, 45, 76, 78). Jabbour et al., reported a significant decrease in glucose concentrations after an acute (1 hour) session of low frequency NMES (8 Hz) in a middle-aged population with T2D and reported to be tolerable by all participants (45). Several studies that used low frequency NMES, reported increases in glucose utilization with variable NMES intensities (29, 31, 45, 50–52, 56–59, 61, 62, 70, 73, 75–79). These findings are also supported by Joubert et al., 2015 (76), demonstrating that after a single 25-minute session of low frequency (35 Hz) NMES, a significant increase was reported in glucose uptake as measured by the hyperinsulinemic euglycemic clamp in a population with T2D. On the other hand, when a chronic high frequency protocol was applied to individuals with T2D, no significant changes to glucose uptake was reported. It was also noted that participants were unable to tolerate NMES intensities above 40 mA (approximately 10% of maximum voluntary contraction) (69). In rehabilitation settings, NMES is commonly used to aid in completion of functional tasks and therefore intensity of NMES varies in general practice. Previous studies adjusted the treatment intensity to achieve the desired outcome of visible muscle contractions, cadence count, or amount of oxygen consumption (23, 50, 70, 79). Present literature also supports the improvement in glycemic control when high frequency NMES was used in populations with T2D or SCI. High frequency NMES has been shown to be effective in majority of the studies (23, 30, 59, 63, 64, 66, 72, 74), except for two studies (65, 69) that have not only used a low intensity, but also used NMES only once a week, which might explain the lack of improvements in glycemic control reported by these two studies. Moreover, one of these studies utilized the Borg rates of perceived exertion of 5-6 on a 10-point scale that relied on the participants rating/selecting their treatment intensity and could have led to inconsistencies and varied intensities throughout the study (65). As previously mentioned, the other study utilized a 40 mA intensity which is equivalent to ~10% of a maximum voluntary contraction (69). Limited studies have specifically investigated the role of NMES frequency and intensity on glycemic control. Most of the studies that reported improvement in glycemic control have used a duration of 20-30 minutes a session, between 2-3 times per week and 4-8 weeks NMES intervention (see Table, **Supplementary Table 2**).

Taken together, the present evidence suggests that regardless of frequency and intensity used, NMES is effective in improving glycemic control. NMES used between 2-3/week with a minimum duration of 2 weeks seems to effectively improve glycemic control in populations with insulin resistance or those who are unable to adhere to regular exercise or physical activity.

### Clinical implications

The main clinical applications for NMES have focused on rehabilitation for muscle strengthening, maintenance of muscle mass and strength during prolonged periods of immobilization, selective muscle training, and control of edema (21). Using these same endpoints, NMES is being used for body contouring in aesthetic medicine to selectively increase muscle mass and tone in muscle groups of the body (66, 84). NMES is also being used to increase blood flow in the extremities to avoid blood clots and promote wound healing (83). Understanding that NMES can improve glycemic control, it has a potential to be incorporated into the multidimensional (diet, exercise, behavior modification, and pharmaceuticals) treatment of obesity, metabolic syndrome, and type 2 diabetes as a complement or when traditional exercise modalities are not feasible. Further research will be necessary to determine the ideal treatment protocols.

Our study is not without limitations. First, our meta-analysis is limited by small number of randomized controlled trials that has been conducted to determine the effects of NMES on glycemic control. However, this is the first comprehensive review that has reviewed all existing literature to address the effectiveness of NMES on improving insulin sensitivity. The systematic review with meta-analysis strongly indicates the effectiveness of NMES in improving glycemic control and insulin sensitivity. Second, two studies included in this meta-analysis incorporated exercise in addition to NMES treatment (72, 79). However, the outcome does not change when those two studies were excluded from the analysis. Third, most of the studies that utilized NMES were predominantly conducted in population with T2D and SCI. Therefore, present evidence may not be translatable to all population. Future studies should investigate the effectiveness of NMES in healthy population.

In summary, this is the first comprehensive systematic review with meta-analysis to determine the effects of NMES on glycemic control. Our analysis strongly suggests that NMES can effectively improve glycemic control, mainly in population with T2D and those incapable of doing regular traditional exercises (people with SCI). Present literature is not adequate to conclude the effects of NMES on substrate utilization or body composition. Our results strongly suggest the promising potential of NMES use as an alternative therapeutic to improve glycemic control. This systematic review and meta-analysis will serve as a groundwork for many future studies that employ the use of NMES and its evidence-based effectiveness on improving glycemic control in populations with impairments in glycemic control and/or physical limitations.

## Data availability statement

The original contributions presented in the study are included in the article/**Supplementary Material**, further inquiries can be directed to the corresponding author/s.

## Author contributions

MS, MG, AM, and SS did the initial search and selected articles for the study. JA did additional search. All reviewers assessed the selected articles for inclusion. All authors contributed to the writing and revision of the article and approved the submitted version.
