# Supplementary figures and images for: Effects of neuromuscular electrical stimulation on glycemic control: a systematic review and meta-analysis
Source: Front Endocrinol (Lausanne). 2023 Jul 31;14:1222532. doi: 10.3389/fendo.2023.1222532 (PMC10424918; doi:10.3389/fendo.2023.1222532)

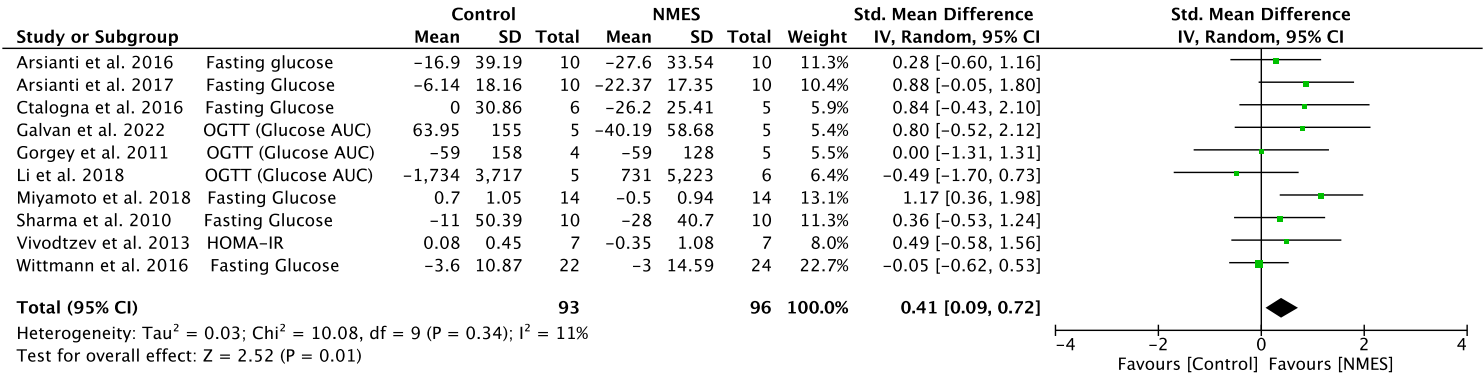

Supplement: Supplementary file 1 [file Image_1.pdf]
