# Supplementary material for: Effects of neuromuscular electrical stimulation on glycemic control: a systematic review and meta-analysis
Source: Front Endocrinol (Lausanne). 2023 Jul 31;14:1222532. doi: 10.3389/fendo.2023.1222532 (PMC10424918; doi:10.3389/fendo.2023.1222532)
Supplement: Supplementary file 2 [file Table_1.docx]

## Supplementary Table 1

**Keyword/Meshword Search Strategies**

*Electronic databases: MEDLINE (PubMed), EMBASE, Cochrane Library, Google Scholar, and Web of science.*

Neuromuscular electrical stimulation OR

NMES OR

electromyostimulation OR

EMS OR

electrical stimulation OR

electrical muscle stimulation OR

electrical pulse stimulation OR

EPS

AND

blood glucose OR

insulin sensitivity OR

glucose OR

insulin OR

metabolic health OR

metabolic improvement OR

metabolic

AND

muscle
