# Supplementary material for: Effects of neuromuscular electrical stimulation on glycemic control: a systematic review and meta-analysis
Source: Front Endocrinol (Lausanne). 2023 Jul 31;14:1222532. doi: 10.3389/fendo.2023.1222532 (PMC10424918; doi:10.3389/fendo.2023.1222532)
Supplement: Supplementary file 3 [file Table_2.docx]

Supplementary Table 2. Population characteristics of included studies

| **Study** | **Number of participants** | **Study population** | **NMES  Intervention** | **NMES duration (min)** | **NMES frequency (Hz)** | **NMES pulse width (µs)** | **NMES Intensity** | **Method to  measure  IS** | **Glycemic control outcome** | **Body composition (Methods)** | **Substrate utilization (Methods)** |
| --- | --- | --- | --- | --- | --- | --- | --- | --- | --- | --- | --- |
| ***Arsianti et al. 2016** | N=20  NMES=10  Passive Stretching=10 | Men and women with  T2D (≥55 years old) | 3x/week for 4 weeks | 30 | Low 20 | 200 | 60 mA | Fasting BG | BG: Decreased | Not measured | Not measured |
| ***Arsianti et al. 2017** | N=20  NMES=10  Control=10 | Men and women with T2D (≥35 years old) | 3x/week for 4 weeks | 30 | Low 20 | 200 | NA | Fasting BG | BG: Decreased | Not measured | Not measured |
| ***Catalogna et al. 2016** | N=11  NMES=5  Control=6 | Men and women with T2D  (45-75 years old) | 7x/week for 2 weeks | 5 | Low 1.33 / burst mode of 16 | 150 | 5-10 mA | MGTT | BG: Decreased  Postprandial  Glucose: Decreased | Not measured | Not measured |
| **Chen et al. 2022** | N=9  NMES=9  Control=NA | Healthy males  (18-65 years old) | One session (Acute) | 120 | Low 20 |  | Not reported | OGTT | Glucose AUC: No change | Not measured | EE: Decreased  RER: Decreased |
| **Chilibeck et al. 1999** | N=5  NMES=5  Control=NA | Middle-aged men and women with SCI (31-50 years old) | 3x/week for 8 weeks | 30 | Low 30 | NA | 10-140 mA | OGTT | IS index: Increased | Not measured | Not measured |
| **Cohen et al. 2022** | N=13  NMES=13  Control=NA | Young healthy males and females  (<35 years old) | One session (Acute) | 30 | Low 5 | 200 | Not reported | OGTT | BG: No change | Not measured | VO2: Increased  RER: Increased |
| **Erickson et al. 2017** | N=14  NMES=14  Control=NA | Men and women with  SCI (30-63 years old) | 3-5x/week for 16 weeks | 10-75 | Low 2-7 | 200 | Visual vigorous muscle contraction | OGTT | BG: No change  HOMA-IR: No change  HbA1c: Decreased | Bilateral quadriceps muscle: No change (MRI) | Not measured |
| ***Galvan et al. 2022** | N=10  NMES=5  Control=5 | Overweight/. obese men and women  (18-54 years old) | 3x/week for 4 weeks | 30 | High 50 | 300 | Max tolerable | OGTT | BG: No change  Glucose AUC: Decreased | BW: No change  BMI: No change  FM: No change  LM: No change (DXA) | RQ: No change  (Indirect Calorimetry) Lactate: Increased |
| **Giggins et al. 2017** | N=13  NMES=13  Control=NA | Men with T2D  (45.1-58.9 years old) | 6x/week for 8 weeks | 60 | Low 4-19 | 760 | Max tolerable | Fasting BG | BG: Decreased  HbA1c: No change | BW: Decreased  Body fat: Decreased  LM: No change (DXA) | Not measured |
| **Gorgey et al. 2011** | N=9  NMES  +Diet=5  Diet =4 | Men with SCI  (26-44 years old) | 2x/week for 12 weeks | NA | Low 30 | 450 | Raised until visible contraction | OGTT | Glucose AUC: Decreased  Insulin AUC: No change  HOMA-IR: No change | BW: No change  BMI: No change  FM: No change  LM: No change  CSA: Increased (DXA) | Not measured |
| **Griffin et al. 2007** | N=18  NMES=18  Control=NA | Men and women with SCI (25-57 years old) | 2-3x/week for 10 weeks | 30 | High 50 | NA | Increased to promote a cadence of 49 revolutions | OGTT | BG: Decreased  Insulin level: Decreased | BW: Increased  FM: No change  LM: Increased (DXA) | Not measured |
| **Guzman et al. 2019** | N=32  NMES=16  Control=16 | Men and women with T2D  (18-30 years old) | Week 1 was control condition followed by 1x/week for 34 weeks | 20 | Low 5,10, and High 50 | 400 | Max tolerable | Fasting BG | BG: Decreased | Not measured | Not measured |
| **Hamada et al. 2003** | N=14  NMES=14  Control=NA | Young healthy males (24.2-25.4 years old) | One session (Acute) | 20 | Low 20 | 0.2 | Limit of 80 mA | Clamp | GDR: Increased | Not measured | RER: Increased  Lactate: Increased  VO2: Increased (Indirect Calorimetry) |
| **Hamada et al. 2004** | N=8  NMES=8  Control=NA | Young healthy males (22.8-24 years old) | One session (Acute) | 20 | Low 20 | 0.2 | Limit of 80 mA | Clamp | GDR: Increased | Not measured | RER: Increased  Lactate: Increased  VO2: Increased (Indirect Calorimetry) |
| **Hioki et al. 2021** | N=22  NMES=12  Control=10 | Active, non-obese males and females | One session  (Acute) | 30 | Low 30 | 300 | Not reported | Fasting BG | BG: Decreased | Not measured | Not measured |
| **Hoshiai et al. 2020** | N=19  NMES=19  Control=NA | Healthy males and females (22-33 years old) | One session (Acute) | 20 | Low 20 | 100 | Max tolerable | Fasting BG | BG: Decreased | Not measured | Blood lactate: increased |
| **Jabbour et al. 2015** | N=8  NMES=8  Control=NA | Middle-aged men and women with T2D (39-65 years old) | One session (Acute) | 60 | Low 8 | 200 | Max tolerable | OGTT | BG: Decreased | Not measured | Not measured |
| **Jeon et al. 2002** | N=7  NMES=7  Control=NA | Middle-aged men and women with SCI (30-53 years old) | 3x/week for 8 weeks | 30 | Low 30 | NA | 10-140 mA | OGTT / Clamp | BG: Decreased | Not measured | Not measured |
| **Jeon et al. 2010** | N=8  NMES=6  Drop out=2 | Middle-aged men with SCI  (24-56 years old) | 3-4x/ week for 12 weeks | 2 | NA | NA | Max tolerable | Fasting BG | BG: Decreased  HbA1c: No change | BW: Decreased FM: Decreased  LM: No change (DXA) | Not measured |
| **Joubert et al. 2015** | N=18  NMES=18  Control=NA | Men and women with T2D  (49-68 years old) | One session (Acute) followed by 6x/week | 25 | Low 35 | 350 | Max tolerable | Clamp | Insulin sensitivity index: Increased | Not measured | Energy Expenditure: No change |
| **Kimura et al. 2010** | N=14  NMES=14  Control=NA | Middle-aged obese and pre-obese men  (42.1-47.7 years old) | One session (Acute) | 20 | Low 4 | 0.2 | Max tolerable | Meal Tolerance Test | BG: Decreased  Glucose AUC: Decreased  Insulin AUC: Decreased | Not measured | RQ: No change  Lactate: Increased (Indirect Calorimetry) |
| ***Li et al. 2018** | N=11  NMES=6  High protein diet=5 | Middle-aged men and women with SCI (37-58 years old) | 3x/week for 8 weeks | 30 | High 50 | 450 | NA | OGTT | BG: Decreased  Insulin AUC: Decreased  Fasting insulin: No change  Matsuda Index: No change  HOMA-IR: No change  Glucose AUC: No change | Body Mass: Decreased FM: Decreased LM: No change  Android Fat Mass: Trend for Decrease (DXA) | Not measured |
| **Mahoney et al. 2005** | N=5  NMES=5  Control=NA | Men with SCI  (30.7-40.5 years old) | 2x/week for 12 weeks | NA | Low 30 | 450 | NA | OGTT | BG: Trend decreased  Insulin: No change | Quadriceps femoris muscle CSA: Increased (MRI) | Not measured |
| **Man et al. 2011** | N=52  TENS=26  Control=26 | Middle-aged women  (35-75 years old) | One session (Acute) | 30 | Low 15 | NA | 10mA | Fasting BG and Insulin | BG: Decreased  HOMA-IR: Increased | Not measured | Not measured |
| **Miyamoto et al. 2012** | N=11  NMES=11  Control=NA | Middle-aged men with T2D  (54.3-59.7 years old) | One session (Acute) | 30 | Low 4 | 0.2 | Max tolerable | Meal Tolerance Test | BG: Decreased  Insulin Level: No change | Not measured | RQ: Increased  Lactate: Increased  VO2: Increased (Indirect Calorimetry) |
| **Miyamoto et al.**  **2015** | N=18  Men=10  Women=8  Control=18 | Men and women with T2D  (47.1-75.8 years old) | One session (Acute) | 30 | Low 4 | 0.2 | 6.0 ml/kg/min oxygen consumption | Meal Tolerance Test | BG: Decreased  HbA1c: No change  HOMA-IR: No change | Not measured | RQ: Increased  Lactate: Increased  VO2: Increased (Indirect Calorimetry) |
| ***Miyamoto et al. 2018** | N=28  NMES=14  Control=14 | Elderly men with T2D (60.2-66.2 years old) | 5x/week for 8 weeks | 40 | Low 4 | 0.2 | Max tolerable | Fasting BG | BG: Decreased  HbA1c: No change | BW: No change  BMI: No change %BF: Decrease  LM: No change (BIA) | Not measured |
| **Mohr et al. 2001** | N=10  NMES=10  Control=NA | Middle-aged men and women with SCI (27-45 years old) | 3x/week for 12 months followed by 1x/week for 6 months | 30 | Low 30 | 350 | 120 mA | OGTT / Clamp | BG: No change  IS: Increased | Not measured | Not measured |
| **Poole et al. 2005** | N=5  NMES=5  Control=NA | Middle-aged men and women with T2D (21-55 years old) | 1x/day for 12 weeks | 240 | High 50 | NA | 40 mA | OGTT / Clamp | BG: No change  Insulin level: No change  HbA1c: No change | BW: No change BMI: No change FM: No change LM: No change (BIA) | Energy expenditure: Increased |
| ***Sharma et al. 2010** | N=20  NMES=10  Control=10 | Men and women with T2D  (>55 years old) | 3x/week for 2 weeks | 40 | High 50 | NA | Max tolerable | Fasting BG | BG: Decreased | Not measured | Not measured |
| **Van Buuren et al. 2015** | N=15  NMES=15  Control=NA | Elderly men and women with T2D (57.91-65.5 years old) | 2x/week for 10 weeks | 20 | High 80 | NA | Max tolerable | Fasting BG | BG: Decreased  HbA1c: Decreased | BW: No change FM: No change LM: No change (BIA) | Not measured |
| ***Vivodtzev et al. 2013** | N=14  NMES=7  Control=7 | Men and women with Cystic fibrosis  (21-43 years old) | 4x/week for 6 weeks | 30 | Low 35 for 2 weeks followed by High 50 for 4 weeks | 400 | Max tolerable | Fasting BG | BG: Decreased  HOMA-IR: Decreased | Mid-Thigh circumference and quadricep strength: Increased | Not measured |
| **Wall et al. 2012** | N=6  NMES=6  Control=NA | Elderly men with T2D (68-72 years old) | One session (Acute) | 60 | High 60 | 500 | Max tolerable | Fasting BG | BG: Decreased  Insulin level: No change | Not measured | Not measured |
| ***Wittmann et al. 2016** | N=75  NMES=24  NMES+Diet=21  Control=22  Drop out=8 | Elderly women with Sarcopenic obesity (≥70 years old) | 1x/week for 26 weeks | 11-20 | High 85 | 350 | Borg rates of perceived exertion of 5-6 on a 10 point scale | Fasting BG | BG: No change | Waist Circumference: Decreased (DXA) | Not measured |

| **List of Abbreviations**  NMES: Neuromuscular electrical stimulation  IS: Insulin sensitivity  T2D: Type 2 diabetes mellitus  SCI: Spinal cord injury  OGTT: Oral Glucose Tolerance Test  MGTT: Meal Glucose Tolerance Test  Clamp: Hyperinsulinemic Euglycemic Clamp  HOMA-IR: Homeostatic Model Assessment of Insulin  Resistance  HbA1c: Glycated Hemoglobin | BG: Blood glucose  GDR: Glucose Disposal Rate  BW: Body weight  FM: Fat mass  BF: Body fat  LM: Lean mass  CSA: Cross sectional area  MRI: Magnetic Resonance Imaging  DXA: Dual energy X-ray Absorptiometry  BIA: Body Impedance Analysis  RER: Respiratory Exchange Ratio | RQ: Respiratory Quotient  VO2: Oxygen Consumption  NA: Not Applicable  Min: Minute  Hz: Hertz  µs: Microsecond  *: Included in meta-analysis |
| --- | --- | --- |
